# Methotrexate, Doxorubicin, and Cisplatin (MAP) Plus Maintenance Pegylated Interferon Alfa-2b Versus MAP Alone in Patients With Resectable High-Grade Osteosarcoma and Good Histologic Response to Preoperative MAP: First Results of the EURAMOS-1 Good Response Randomized Controlled Trial

**DOI:** 10.1200/JCO.2014.60.0734

**Published:** 2015-06-08

**Authors:** Stefan S. Bielack, Sigbjørn Smeland, Jeremy S. Whelan, Neyssa Marina, Gordana Jovic, Jane M. Hook, Mark D. Krailo, Mark Gebhardt, Zsuzsanna Pápai, James Meyer, Helen Nadel, R. Lor Randall, Claudia Deffenbaugh, Rajaram Nagarajan, Bernadette Brennan, G. Douglas Letson, Lisa A. Teot, Allen Goorin, Daniel Baumhoer, Leo Kager, Mathias Werner, Ching C. Lau, Kirsten Sundby Hall, Hans Gelderblom, Paul Meyers, Richard Gorlick, Reinhard Windhager, Knut Helmke, Mikael Eriksson, Peter M. Hoogerbrugge, Paula Schomberg, Per-Ulf Tunn, Thomas Kühne, Heribert Jürgens, Henk van den Berg, Tom Böhling, Susan Picton, Marleen Renard, Peter Reichardt, Joachim Gerss, Trude Butterfass-Bahloul, Carol Morris, Pancras C.W. Hogendoorn, Beatrice Seddon, Gabriele Calaminus, Maria Michelagnoli, Catharina Dhooge, Matthew R. Sydes, Mark Bernstein

**Affiliations:** Stefan S. Bielack, Klinikum Stuttgart–Olgahospital, Stuttgart; Mathias Werner, Helios Klinikum Emil von Behring; Per-Ulf Tunn, Helios Klinikum Berlin-Buch, Berlin; Knut Helmke, Altonaer Kinderkrankenhaus, Hamburg; Heribert Jürgens, Gabriele Calaminus, Joachim Gerss, and Trude Butterfass-Bahloul, Universitätsklinikum Münster, Münster; Peter Reichardt, Klinik für Interdisziplinäre Onkologie, Bad Saarow, Germany; Sigbjørn Smeland and Kirsten Sundby Hall, Oslo University Hospital; Kirsten Sundby Hall, Norwegian Radium Hospital, Oslo, Norway; Jeremy S. Whelan, University College London Hospitals; Gordana Jovic, Jane M. Hook, and Matthew R. Sydes, University College London; Beatrice Seddon and Maria Michelagnoli, University College Hospital, London; Bernadette Brennan, Christie Hospital and Royal Manchester Children's Hospital, Manchester; Susan Picton, Leeds University Hospital, Leeds, United Kingdom; Neyssa Marina, Stanford University Medical Center; Claudia Deffenbaugh, Lucille Salter Packard Children's Hospital, Palo Alto; Mark D. Krailo, Children's Oncology Group, Arcadia, CA; Mark Gebhardt and Allen Goorin, Dana-Farber Cancer Institute; Mark Gebhardt and Lisa A. Teot, Children's Hospital Boston, Boston, MA; Zsuzsanna Pápai, National Medical Center, Budapest, Hungary; James Meyer, Children's Hospital of Philadelphia and University of Pennsylvania School of Medicine, Philadelphia, PA; Helen Nadel, British Columbia Children's Hospital and University of British Columbia, Vancouver, British Columbia; Mark Bernstein, Dalhousie University, Halifax, Nova Scotia, Canada; R. Lor Randall, University of Utah, Salt Lake City, UT; Rajaram Nagarajan, Cincinnati Children's Hospital Medical Center, Cincinnati, OH; G. Douglas Letson, H. Lee Moffit Cancer Center and Research Institute, Tampa, FL; Daniel Baumhoer, Universitätsspital Basel; Thomas Kühne, University Children's Hospital Basel, Basel, Switzerland; Leo Kager, St Anna's Children Hospital; Reinhard Windhager, Medical University of Vienna, Vienna, Austria; Ching C. Lau, Baylor College of Medicine, Houston, TX; Hans Gelderblom and Pancras C.W. Hogendoorn, Leiden University Medical Center, Leiden; Peter M. Hoogerbrugge, Radbound University Nijmegen Medical Centre, Nijmegen; Henk van den Berg, Emma Children's Hospital–Academic Medical Centre, Amsterdam, the Netherlands; Paul Meyers and Carol Morris, Memorial Sloan Kettering Cancer Center, New York; Richard Gorlick, Montefiore Medical Center, Bronx, NY; Mikael Eriksson, Skane University Hospital, Lund, Sweden; Paula Schomberg, Mayo Clinic, Rochester, MN; Tom Böhling, University of Helsinki and Hospital District of Helsinki and Uusimaa Laboratoriot, Helsinki, Finland; Marleen Renard, University Hospital Leuven, Leuven; and Catharina Dhooge, University Hospital Ghent, Ghent, Belgium.

## Abstract

**Purpose:**

EURAMOS-1, an international randomized controlled trial, investigated maintenance therapy with pegylated interferon alfa-2b (IFN-α-2b) in patients whose osteosarcoma showed good histologic response (good response) to induction chemotherapy.

**Patients and Methods:**

At diagnosis, patients age ≤ 40 years with resectable high-grade osteosarcoma were registered. Eligibility after surgery for good response random assignment included ≥ two cycles of preoperative MAP (methotrexate, doxorubicin, and cisplatin), macroscopically complete surgery of primary tumor, < 10% viable tumor, and no disease progression. These patients were randomly assigned to four additional cycles MAP with or without IFN-α-2b (0.5 to 1.0 μg/kg per week subcutaneously, after chemotherapy until 2 years postregistration). Outcome measures were event-free survival (EFS; primary) and overall survival and toxicity (secondary).

**Results:**

Good response was reported in 1,041 of 2,260 registered patients; 716 consented to random assignment (MAP, n = 359; MAP plus IFN-α-2b, n = 357), with baseline characteristics balanced by arm. A total of 271 of 357 started IFN-α-2b; 105 stopped early, and 38 continued to receive treatment at data freeze. Refusal and toxicity were the main reasons for never starting IFN-α-2b and for stopping prematurely, respectively. Median IFN-α-2b duration, if started, was 67 weeks. A total of 133 of 268 patients who started IFN-α-2b and provided toxicity information reported grade ≥ 3 toxicity during IFN-α-2b treatment. With median follow-up of 44 months, 3-year EFS for all 716 randomly assigned patients was 76% (95% CI, 72% to 79%); 174 EFS events were reported (MAP, n = 93; MAP plus IFN-α-2b, n = 81). Hazard ratio was 0.83 (95% CI, 0.61 to 1.12; *P* = .214) from an adjusted Cox model.

**Conclusion:**

At the preplanned analysis time, MAP plus IFN-α-2b was not statistically different from MAP alone. A considerable proportion of patients never started IFN-α-2b or stopped prematurely. Long-term follow-up for events and survival continues.

## INTRODUCTION

Osteosarcoma is the most frequent primary sarcoma of bone, primarily diagnosed in adolescents and young adults; however, it is rare overall, with only two to three affected individuals per million person-years. Most recent regimens have included several weeks of preoperative chemotherapy, followed by surgery and several months postoperative chemotherapy.^1^ Reported outcomes have been similar internationally and have shown little improvement over previous decades.^2,3^ Histologic response to preoperative chemotherapy is an important prognostic factor. A good histologic response is usually classified as < 10% viable tumor in the resected specimen. Good responders have had better 5-year survival than poor responders (75% to 80% *v* 45% to 55%).^1,4^

Four international osteosarcoma groups with a history of successfully conducted clinical trials^5–9^ formed the European and American Osteosarcoma Study Group (EURAMOS)^10,11^: the Children's Oncology Group (COG), Cooperative Osteosarcoma Study Group (COSS), European Osteosarcoma Intergroup (EOI), and Scandinavian Sarcoma Group (SSG). The EURAMOS-1 trial established large-scale multinational cooperation in clinical trials for osteosarcoma.^11^ MAP (methotrexate, doxorubicin, and cisplatin) chemotherapy was accepted as standard.^8,12^ Intensified salvage chemotherapy was investigated in poor responders. On the basis of preclinical and clinical evidence,^13^ we decided to investigate the value of maintenance treatment with interferon alfa (IFN-α) in good responders.

Maintenance treatment is well established in acute lymphoblastic leukemia^14^ and is being investigated in sarcomas.^15^ IFN-α has antiproliferative, differentiation-inducing, apoptotic, and antiangiogenic properties, and its clinical activity has been demonstrated in several cancers, including as postchemotherapy maintenance.^13,16–19^ It has been associated with activity against osteosarcomas (some of which have expressed IFN-α/β receptor^20^) in vitro, in animal models, and in patients with metastatic disease.^13^ Most notably, single-institution treatment of 89 consecutive patients with semipurified leukocyte IFN-α as the only adjuvant treatment after surgery resulted in 10-year metastasis-free and sarcoma-specific survival rates of 39% and 43%, respectively.^21^ On the basis of this rationale, we aimed to test IFN-α as maintenance treatment in osteosarcoma.^13^ The objective of our random assignment was to examine whether addition of a pegylated formulation of interferon alfa-2b (IFN-α-2b) as maintenance therapy after postoperative MAP would improve outcomes, with event-free survival (EFS) as the primary outcome measure.

## PATIENTS AND METHODS

### Setting

EURAMOS-1 was an open-label phase III randomized controlled trial (RCT) for patients with localized or metastatic high-grade osteosarcoma considered suitable for complete surgical resection. Eligibility for registration has been described previously.^10,11^ Key criteria were localized or metastatic high-grade osteosarcoma of an extremity or the axial skeleton (with exception of craniofacial sites), with all disease sites potentially amenable to complete surgical resection, and age ≤ 40 years.

All patients received induction MAP followed by surgery of the primary. Thereafter, patients age ≥ 5 years who had completed two cycles of induction MAP, had undergone macroscopically complete resection of their primary tumor, had < 10% viable tumor on histologic response assessment, and had no evidence of disease progression were eligible for the good response random assignment. Histologic response assessment was conducted locally before random assignment and later confirmed by a trial reference pathologist. Random assignment had to be performed < 35 days after surgery. Patients age < 5 years at potential random assignment were excluded from random assignment because of reports of neurologic complications in young children receiving IFN-α for other diseases.^22^ Participants and/or their legal guardians, as appropriate, provided written informed consent to registration and random assignment. Regulatory and ethics approvals were obtained according to national requirements.

### Trial Treatments and Procedures

**Fig 1.**
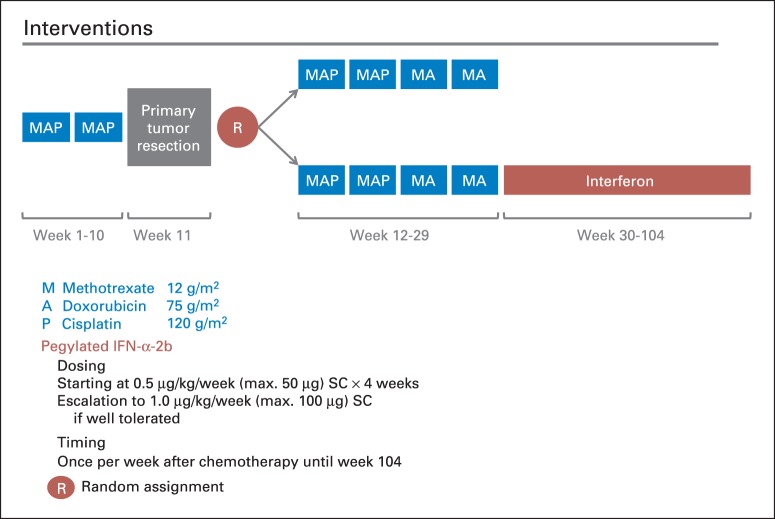
Treatment scheme. IFN-α-2b, interferon alfa-2b; MA, methotrexate, doxorubicin; MAP, methotrexate, doxorubicin, and cisplatin; R, random assignment; SC, subcutaneously.

Induction MAP (weeks 1 to 10) comprised two 5-week cycles of doxorubicin 75 mg/m^2^ of body-surface area, cisplatin 120 mg/m^2^, and methotrexate 12 g/m^2^, followed by surgery of the primary in week 11. Doxorubicin and cisplatin were administered in weeks 1 and 6 and methotrexate in weeks 4, 5, 9, and 10 (Fig 1, treatment scheme; Data Supplement). Up to two additional doses of methotrexate were permitted preoperatively if surgery had to be delayed. The protocol (Data Supplement) contained detailed guidance on mandatory tests and requirements for each treatment cycle, supportive care, and dose adjustments. If present, primary metastases were to be surgically removed in weeks 11 to 20.

After histologic assessment of the resected tumor, consenting patients were randomly assigned in a one-to-one ratio to four postoperative cycles of MAP (weeks 12 to 29; cisplatin omitted in last two cycles) or to the same regimen followed by maintenance pegylated IFN-α-2b (Fig 1). Treatment allocation was performed using concealed permuted blocks with three stratification factors: trial group (COG, COSS, EOI, or SSG), location of tumor (proximal femur or proximal humerus *v* other limb *v* axial skeleton), and presence of metastases (no *v* yes or possible). Lung metastases, detected by spiral computed tomography scanning, were considered certain if there were three or more lesions ≥ 5 mm in maximum diameter or a single lesion ≥ 1 cm. Scans of patients registering metastatic disease with fewer or smaller lesions were classified as possible metastatic disease. Patients were randomly assigned centrally through the Medical Research Council Clinical Trials Unit (COSS, EOI, and SSG) or COG.

Subcutaneous IFN-α-2b was planned weekly from week 30 to 104 at 0.5 μg/kg per week (maximum, 50 μg) for 4 weeks and increased to 1.0 μg/kg per week (maximum, 100 μg) thereafter if no flu-like symptoms worse than Common Toxicity Criteria for Adverse Events (version 3.0)^23^ grade 2 or other toxicities worse than grade 1 were experienced.

### Assessments

During MAP treatment, clinical and toxicity assessments were performed before each drug administration. During IFN-α-2b, patients were monitored twice per week for 8 weeks and once or twice per month thereafter. Adverse events were graded according to the Common Toxicity Criteria for Adverse Events (version 3.0)^23^ and reported centrally as the maximum grade during pre- and postoperative chemotherapy and maximum grade per 3-month period during IFN-α-2b. Toxicity was assessed in each patient until trial treatment was stopped. Late toxicity throughout follow-up was collected at COSS, EOI, and SSG.

All patients were assessed for local and distant recurrence at predefined intervals by physical examination and radiography of the chest and primary site. Radiographically detected relapse was also imaged by computed tomography, magnetic resonance imaging, and/or bone scans and, if appropriate, confirmed by histology. Patients were observed regularly for ≥ 5 years after treatment (Data Supplement).

### Statistical Analyses

The primary outcome measure was EFS, defined as time from random assignment until a first event (local recurrence, new metastatic disease, progression of primary metastatic disease, secondary malignancy, or death) or censoring at last contact. Secondary outcome measures included: overall survival (OS; time from random assignment until death resulting from any cause or last contact), short- and long-term toxicities, and quality of life, which will be the topic of separate analyses.

To detect absolute improvements of 10% from 70% to 80% in 3-year EFS (hazard ratio [HR], 0.63 in favor of IFN-α-2b) with two-sided 5% significance level and 80% power required ≥ 147 EFS events.^24^ The same applied to an improvement in 5-year OS from 70% to 80%, requiring ≥ 147 deaths in the longer term. The initial plan to register 1,400 patients (to randomly assign 1,260 [good responders, n = 567; poor responders, n = 693]) was revised to approximately 2,000 patients because of a lower randomization rate and relatively fewer poor responders than anticipated.^11^ Interim data were reviewed annually by an independent data monitoring committee and could have been reported early if either *P* ≤ .001 for EFS^25,26^ or severe IFN safety issues were identified.

A prespecified subgroup of patients with localized disease comprised those without definitive metastases at registration. To detect a 10% improvement from 75% to 85% in 3-year EFS and 5-year OS (HR, 0.56) with two-sided 5% significance and 80% power required 98 events.

The primary analysis used intention-to-treat principles. The Kaplan-Meier method was used to estimate survival functions, log-rank tests for differences between survival curves, and Cox models (adjusted for stratification factors) to estimate treatment effects, with suitability checked by tests for proportionality of hazards. All comparisons were expressed relative to control, with HR < 1 favoring IFN-α-2b. Consistency of treatment effect was examined using the interaction test (χ^2^ test for heterogeneity) in subgroups defined posthoc: sex, age, site of disease, location on bone, lung metastases, nonlung metastases, and histologic subtype. Median follow-up was calculated using reverse censoring on death.

In a prespecified exploratory analysis, EFS was computed from 23 weeks after starting postoperative chemotherapy, excluding patients who experienced progression before the expected start of IFN-α-2b. IFN-α-2b dose was summarized only for patients who could have completed and reported completing IFN-α-2b by the data freeze (patients registered before November 15, 2010). Analyses were performed using Stata software (versions 12.1 and 13.1; Stata, College Station, TX).

## RESULTS

### Patients

**Fig 2.**
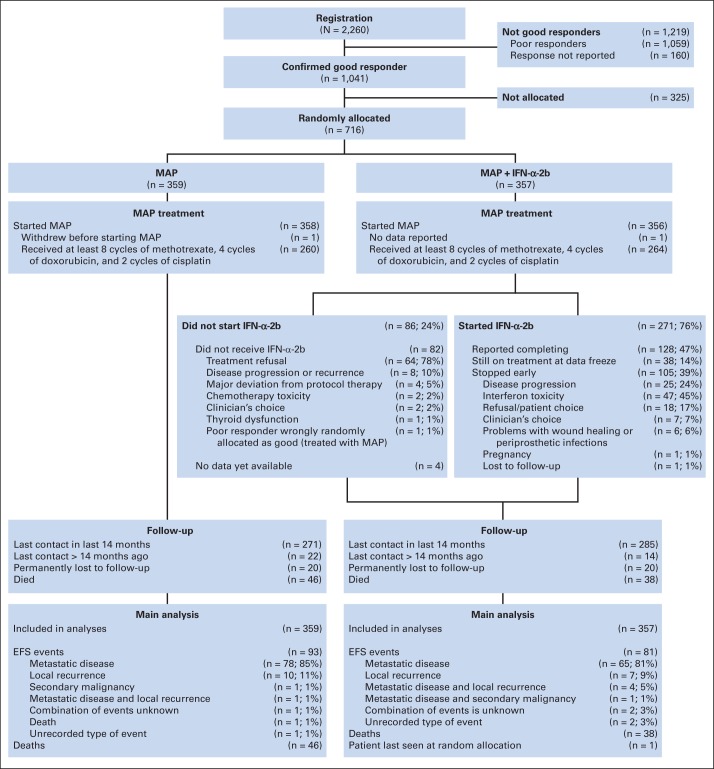
CONSORT diagram. EFS, event-free survival; IFN-α-2b, interferon alfa-2b; MAP, methotrexate, doxorubicin, and cisplatin.

Between April 2005 and June 2011, 2,260 patients were registered from > 300 sites in 17 European, North American, and Australasian countries. The data were frozen on February 15, 2013, because the event target was reached. A total of 1,041 patients were good responders, and 716 (69%) from 246 trial sites were randomly assigned (MAP, n = 359; MAP plus IFN-α-2b, n = 357; Fig 2). COG, COSS, EOI, and SSG randomly assigned 300, 206, 161, and 49 patients, respectively. Table 1 lists registration characteristics for these randomly assigned patients. Median age was 14 years (interquartile range [IQR], 11 to 16), and 421 (59%) were male; 630 (88%) had localized disease, and 86 (12%) had primary metastases; of these, 66 had lung-only, 15 had extrapulmonary-only, and five had both lung and extrapulmonary metastases.

**Table 1.** Patient Characteristics at Registration

Characteristic	MAP (n = 359)		MAP Plus IFN-α-2b (n = 357)		Total (N = 716)	
	No.	%	No.	%	No.	%
Sex						
Male	211	59	210	59	421	59
Female	148	41	147	41	295	41
Age at registration, years						
< 5	0	0	1	0	1	0
5-9	58	16	44	12	102	14
10-19	275	77	288	81	563	79
20-29	22	6	17	5	39	5
> 30	4	1	7	2	11	2
Median	14		14		14	
IQR	11-16		12-16		11-16	
Site of tumor						
Femur	179	50	191	54	370	52
Tibia	113	31	102	29	215	30
Fibula	14	4	20	6	34	5
Humerus	36	10	33	9	69	10
Radius	5	1	5	1	10	1
Ulna	2	1	0	0	2	0
Scapula/clavicle	2	1	1	0	3	0
Pelvis/sacrum	5	1	5	1	10	1
Rib	3	1	0	0	3	0
Location of tumor						
Proximal	156	43	150	42	306	43
Diaphysis	13	4	12	3	25	3
Distal	180	50	189	53	369	52
NA (not long bone)	10	3	6	2	16	2
Pathologic fracture at diagnosis						
No	321	90	308	86	629	88
Yes	37	10	49	14	86	12
Missing	1	NA	0	NA	1	NA
Lung metastases						
No	295	82	288	81	583	81
Possible*	29	8	33	9	62	9
Yes	35	10	36	10	71	10
Other metastases						
No	343	96	348	97	691	97
Possible*	3	1	2	1	5	1
Yes	13	4	7	2	20	3
WHO 2002 classification of osteosarcoma^27^						
Conventional	320	90	322	92	642	91
Telangiectatic	25	7	20	6	45	6
Small cell	2	1	1	0	3	0
High-grade surface	3	1	5	1	8	1
Other	4	1	2	1	6	1
Missing	5	NA	7	NA	12	NA

Abbreviations: IFN-α2b, interferon alfa-2b; IQR, interquartile range; MAP, methotrexate, doxorubicin, and cisplatin; NA, not applicable;

* Possible metastases were collected only by Cooperative Osteosarcoma Study Group, European Osteosarcoma Intergroup, and Scandinavian Sarcoma Group.

Median follow-up was 44 months (IQR, 28 to 58) for MAP and 44 months (IQR, 29 to 58) for MAP plus IFN-α-2b. Twenty patients (6%) in each arm were permanently lost to follow-up. For patients last reported as alive, 94% were seen < 14 months before data freeze.

### Treatment

#### Postoperative MAP.

Postoperative MAP was delivered similarly in both treatment arms. Median standardized postoperative dose of methotrexate was 95 g/m^2^ (target, 96g/m^2^); doxorubicin, 298 mg/m^2^ (target, 300 mg/m^2^); and cisplatin, 239 mg/m^2^ (target, 240 mg/^2^; Data Supplement).

### IFN-α-2b

Of 357 patients randomly assigned to MAP plus IFN-α-2b, 82 (23%) reported not starting; information was missing for four (1%). The most common reason for not starting was treatment refusal (78%; Fig 2). Of the 357 patients, 271 (76%) started IFN-α-2b at median 23 weeks after random assignment. At data freeze, 128 (47%) of these 271 patients reported completing protocol treatment, 105 (39%) of 271 reported stopping early, and 38 (14%) of 271 reported still receiving treatment. Reported reasons for early termination were: toxicity (n = 47; 45%), osteosarcoma progression (n = 25; 24%), refusal or patient choice (n = 18; 17%), clinician decision (n = 7; 7%), problems with wound healing or periprosthetic infections (n = 6; 6%), and other reasons (pregnancy, n = 1; lost, n = 1; Fig 2). Of the 271 patients, 132 (49%) required IFN-α-2b dose reductions or delays. The target cumulative IFN-α-2b dose was 72 μg/kg. In 319 patients who could have completed IFN-α-2b by the data freeze, the observed median dose was 25.8 μg/kg (IQR, 0.5 to 60.0). Of these 319 patients, 240 reported starting IFN-α-2b; among these 240, median dose was 40.0 μg/kg (IQR, 14.5 to 65.0; Data Supplement). Median duration of therapy was 67 weeks (IQR, 25 to 75).

### Efficacy

EFS at 3 years for all 716 randomly assigned patients was 76% (95% CI, 72% to 79%). A total of 174 events were reported (MAP, n = 93; MAP plus IFN-α-2b, n = 81; Fig 2). In both arms, patients' first events mostly included metastases (MAP, n = 79; MAP plus IFN-α-2b, n = 70). Local recurrence was involved in 22 of the 174 first events (MAP, n = 11; MAP plus IFN-α-2b, n = 11). Of these, 17 were isolated local recurrences, and five were combined with distant metastases. One secondary malignancy (acute myeloid leukemia) was reported as a first event (MAP-alone arm). Type of first event was not reported for three patients (MAP, n = 1; MAP plus IFN-α-2b, n = 2).

**Fig 3.**
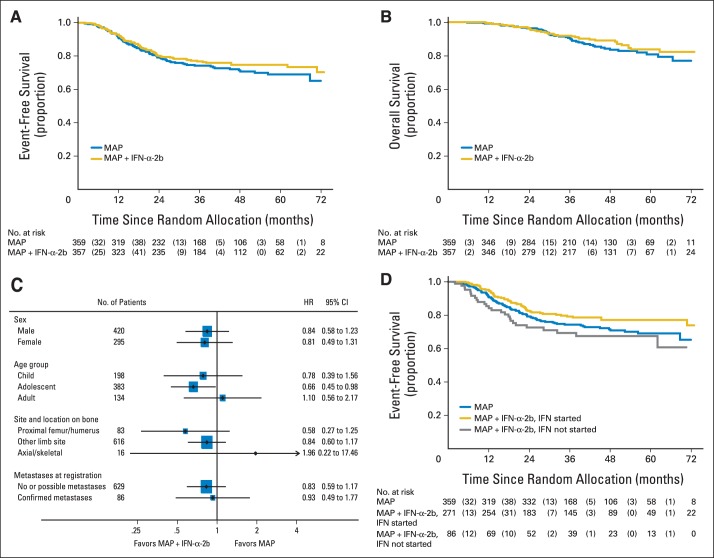
(A) Event-free survival; (B) overall survival; (C) exploratory subgroup analysis; (D) exploratory comparison. Nos. in parentheses in risk tables of parts A, B, and D indicate No. of patients who had an event during the specified time period. HR, hazard ratio. IFN-α-2b, interferon alfa-2b; MAP, methotrexate, doxorubicin, and cisplatin.

Treatment effect for IFN-α-2b was estimated as HR of 0.83 (95% CI, 0.61 to 1.12; *P* = .214). Rates of 3-year EFS for MAP and MAP plus IFN-α-2b were 74% (95% CI, 69% to 79%) and 77% (95% CI, 72% to 81%), respectively (Fig 3A).

In 630 patients with localized disease, 135 EFS events were reported (MAP, n = 72; MAP plus IFN-α-2b, n = 63). The estimated treatment effect was consistent with the whole trial population (HR, 0.83; 95% CI, 0.59 to 1.17; *P* = .284); 3-year EFS estimates were 77% (95% CI, 71% to 82%) and 80% (95% CI, 75% to 84%) for MAP and MAP plus IFN-α-2b, respectively.

A total of 84 deaths were reported (MAP, n = 46; MAP plus IFN-α-2b, n = 38; Fig 3B). This early estimate of survival had an HR of 0.77 (95% CI, 0.50 to 1.19); 5-year OS was 81% (95% CI, 74% to 86%) for MAP and 84% (95% CI, 78% to 88%) for MAP plus IFN-α-2b. Follow-up continues for survival.

### Toxicity

The toxicity of preoperative chemotherapy has previously been reported.^11^ During postoperative MAP, toxicity was mostly hematologic and did not differ by arm (Data Supplement). One patient died as a result of toxicity (cardiomyopathy); worst toxicity was grade 4 for 628 (88%) and grade 3 for 59 (8%) of 716 patients.

With regard to IFN-α-2b, toxicity data were reported for 268 of 271 patients who started IFN-α-2b. No fatal toxicities were reported (Table 2). The worst toxicity during IFN-α-2b was grade 4 for 32 (12%) of 268 patients (primarily hematologic [n = 26] or left ventricular systolic dysfunction [LVSD; n = 4]); grade 3 was worst toxicity for 101 (38%) and grade 1 to 2 for 105 (39%) of 268 patients. Three suspected unexpected serious adverse reactions related to IFN-α-2b were reported: two new cases of LVSD and one knee joint effusion.

**Table 2.** Worst-Grade Toxicities Reported During IFN-α2b Treatment

Toxicity	Worst Grade							
	0		1-2		3		4	
	No.	%*	No.	%*	No.	%*	No.	%*
Any	30	11	105	39	101	38	32	12
Routinely collected toxicities†								
Neutrophils	72	38	39	21	65	34	14	7
Leucocytes	76	29	158	59	25	9	7	3
Platelets	142	53	112	42	6	2	6	2
Mood alteration (depression)	193	75	55	21	8	3	1	0
Fever	156	59	107	40	1	0	1	0
Hemoglobin	131	49	127	48	8	3	0	0
Fatigue	134	50	129	49	3	1	0	0
Cardiac arrhythmia	243	96	7	3	3	1	0	0
Rigor/chills	202	75	64	24	2	1	0	0
Vomiting	236	89	28	11	2	1	0	0
Diarrhea	239	90	25	9	2	1	0	0
Bilirubin	245	92	18	7	2	1	0	0
Weight loss	231	87	35	13	1	0	0	0
Thyroid dysfunction	242	92	20	8	1	0	0	0
Creatinine	250	94	15	6	1	0	0	0
Mucositis	174	94	12	6	0	0	0	0
Other notable serious AEs and toxicities‡								
LVSD	NA	NA	1	0	2	1	4	2
Amylase	NA	NA	0	0	0	0	1	0
Mood alteration (agitation)	NA	NA	0	0	0	0	1	0
Infection (normal neutrophils)§	NA	NA	2	1	6	2	0	0
Flu-like syndrome	NA	NA	12	5	0	0	0	0
Pain (muscle)	NA	NA	4	2	4	2	0	0
Pain (head/headache)	NA	NA	12	5	0	0	0	0
Pain (extremity/limb)	NA	NA	5	2	0	0	0	0

NOTE. Includes all routinely collected toxicities and any other toxicities with reported incidence in ≥ five patients of any grade or of grade ≥ 4 in one patient.

Abbreviations: AE, adverse event; CRF, case report form; IFN-α2b, interferon alfa-2b; LVSD, left ventricular systolic dysfunction; NA, not applicable.

* Based on No. of patients reporting each type of toxicity.

† Routinely collected on CRF.

‡ Spontaneously reported on CRF or as serious AE.

§ Or grade 1 to 2 neutrophils.

From routinely collected long-term toxicity data, seven (4%) of 193 patients receiving MAP and eight (4%) of 199 patients receiving MAP plus IFN-α-2b reported grade 3 to 4 LVSD (Data Supplement). One additional grade 4 LVSD was reported as a serious adverse event during follow-up.

### Exploratory Analyses

Exploratory subgroup analyses found no evidence of heterogeneity in treatment effect (Fig 3C; Data Supplement). An exploratory EFS analysis (Fig 3D) separated patients allocated to MAP plus IFN-α-2b who started IFN-α-2b from those who did not start and compared them with patients allocated to MAP. Patients who did not start their allocated IFN-α-2b seemed to do worse than patients not allocated to IFN-α-2b. The exploratory analysis of EFS computed from 23 weeks after start of postoperative chemotherapy included 702 patients who had not previously experienced progression. HR was 0.83 (95% CI, 0.61 to 1.12), similar to the overall EFS estimate.

## DISCUSSION

We investigated maintenance pegylated IFN-α-2b for patients whose resectable osteosarcomas showed good histologic response to MAP induction chemotherapy. The point estimate of treatment effect showed improved EFS and OS. However, neither was statistically significant, and the CIs were consistent with no effect. No change in practice is indicated by these data.

We were able to ask this question, as well as a parallel question concerning chemotherapy intensification in patients whose osteosarcomas had poor histologic response, only because of the cooperative efforts of four multi-institutional groups.^10,11^ This will provide a framework for future trials.

With an age range up to 40 years and inclusion of patients with resectable axial and/or primary metastatic disease, our study had broader eligibility than many others. However, all patients had their primary tumors resected, and all of these had shown a good response to chemotherapy. The observed 3-year EFS of 76% for the 716 randomly assigned patients meeting our eligibility criteria is in the range of those previously observed for good responders.^4,9,12,28^ Approximately four fifths of first events were exclusively metastatic, and there was no suggestion of an altered distribution of type of event by treatment arm.

Toxicity observed during preoperative MAP was as expected^7^ and did not differ by allocation. Death related to toxicity during postoperative MAP was limited to one case of cardiomyopathy. Nevertheless, most patients reported grade 4 toxicities, mostly hematologic, attesting to the treatment burden of osteosarcoma chemotherapy. As expected,^13,29^ toxicities observed during IFN-α-2b were mainly grade 1 to 2. However, grade 3 and 4 toxicities were reported for one half of patients who started IFN-α-2b, mostly hematologic. Several patients developed signs of cardiac failure during IFN-α-2b. Although we cannot exclude a contribution from IFN-α-2b to this complication, we note these patients had previously received doxorubicin 450 mg/m^2^ and that a similar number of control-arm patients also developed LVSD. Given the high cumulative anthracycline dose, the overall incidence of severe clinical cardiac toxicity in this mainly adolescent population receiving a high cumulative anthracycline dose by continuous infusion, rather than as a bolus, does not seem excessive.^30^

The point estimates of the HR favored IFN-α-2b maintenance for both EFS and OS, but the CIs were consistent with no effect. The observed effect size for EFS (HR, 0.83; 95% CI, 0.61 to 1.12) was similar to that reported for another biologic agent, liposomal muramyl tripeptide phosphatidylethanolamine (HR, 0.80; 95% CI, 0.62 to 1.0),^8^ but smaller than our 0.63 target.

The interpretation of our findings is limited, because approximately one quarter of patients allocated to IFN-α-2b never started it. Furthermore, not all patients continued IFN-α-2b after having started; only 128 of 357 patients reported completing the planned protocol treatment. These issues of initiation and adherence arose even though neither the dose nor schedule of IFN-α-2b nor the duration of treatment was unusual,^16,18,19,31^ and a pegylated preparation was expected to result in fewer adverse effects.^13,16^

One may speculate why the attrition rate for IFN-α-2b was high. Patient choice was the most common reason for non–random assignment and for never starting IFN-α-2b among allocated patients and a common reason for its premature termination. We assume that both previous exposure to 29 protocol weeks of chemotherapy and awareness of a favorable prognosis for good responders affected compliance. A recent RCT of IFN-α maintenance for relapsed lymphoma faced similar abandonment problems. Those researchers concluded that it was not clear whether the absence of a demonstrable advantage reflected a lack of intrinsic activity or indicated the inability to administer an adequate dose of IFN to patients for sustained periods.^32^

At first glance, our observations might call for an as-treated analysis comparing those who initiated IFN-α-2b against the control arm. However, patients allocated to IFN-α-2b who never started the drug fared worse than patients never allocated to receive IFN-α-2b in the first place, for reasons that are currently obscure.

Would a treatment effect have become more obvious if the chosen IFN-α-2b dose had been higher or the treatment period longer? Even in melanoma, where many RCTs of IFN have been performed, evidence supporting a specific IFN dose, duration, or formulation and identification of subsets of patients beyond those with detectable residual disease most likely to benefit remain debatable issues, with no RCT showing additional benefit for treatment extending beyond 12 to 18 months.^19,33^ For osteosarcoma, such evidence is completely absent. The timing of IFN-α-2b therapy is similarly uncertain. Although IFN-α may enhance the sensitivity of osteosarcomas to selected chemotherapeutic agents,^34^ there are no data demonstrating that IFN-α-2b can be safely administered concurrently with MAP and no data indicating that it would be more efficacious.

Was the good responder cohort, with its relatively low recurrence risk, ideal to observe effects of IFN-α-2b? Good responders generally have a lower burden of micrometastatic residual disease (because of chemosensitivity of their osteosarcomas) than poor responders, and IFN may work best in such a context of minimal residual disease. This is exemplified by adjuvant data from melanoma, where IFN activity was confined to a subpopulation with microscopic nodal disease.^19,35^

In conclusion, our collaborative group completed a large prospective RCT in a rare condition within a reasonable timespan. Although the point estimates for EFS and OS favored the intervention—maintenance with pegylated IFN-α-2b—the CIs of the HRs included 1, and we conclude no difference; the observed effect size for EFS was smaller than targeted. A considerable proportion of patients allocated to IFN-α-2b never started or did not complete treatment with the drug, which complicates interpretation of the efficacy data. Reported toxicity in patients who started IFN-α-2b did not seem excessive.

Although we have reached the target number of EFS events, ongoing follow-up of patients is crucial and will permit the planned analysis of OS. The current EFS results, reported at the protocol-defined analytic end point, do not support the routine use of IFN-α-2b maintenance after standard chemotherapy for osteosarcoma.

## Glossary Terms

cisplatin: an inorganic platinum agent (cis-diamminedichloroplatinum) with antineoplastic activity. Cisplatin forms highly reactive, charged, platinum complexes, which bind to nucleophilic groups such as GC-rich sites in DNA, inducing intrastrand and interstrand DNA cross-links as well as DNA-protein cross-links. These cross-links result in apoptosis and cell growth inhibition. Carboplatin and oxaliplatin are other members of this class.
Cox proportional hazards regression model: a statistical model for regression analysis of censored survival data, examining the relationship of censored survival distribution to one or more covariates. This model produces a baseline survival curve, covariate coefficient estimates with their standard errors, risk ratios, 95% CIs, and significance levels.
IFN-α-2b (interferon-alfa-2b): recombinant interferon alfa that is commercially prepared from a bacterial fermentation of *E. coli* bearing an expression vector containing the interferon alfa-2b (*IFN*-α-*2b*) gene from human leukocytes.

## Appendix

### A1. Children's Oncology Group (COG)

#### United States.

Advocate Hope Children's Hospital, Oak Lawn, IL (Dr Ammar Hayani); Advocate Lutheran General Hospital, Park Ridge, IL (Dr Jong Kwon); Albany Medical Center, Albany, NY (Dr Vikramjit Kanwar); Alfred I duPont Hospital for Children, Wilmington, DE (Dr Christopher Frantz); All Children's Hospital, St. Petersburg, FL (Dr Gregory Hale); Ann and Robert H Lurie Children's Hospital of Chicago, Chicago, IL (Dr David Walterhouse); Baptist Hospital of Miami, Miami, FL (Dr Doured Daghistani); Baylor College of Medicine, Houston, TX (Dr Lisa Bomgaars); BI-LO Charities Children's Cancer Center, Greenville, SC (Dr Nichole Bryant); Bronson Methodist Hospital, Kalamazoo, MI (Dr Jeffrey Lobel); Brooke Army Medical Center, Fort Sam Houston, TX (Dr Della Howell); Brooklyn Hospital Center, New York, NY (Dr Swayamprabha Sadanandan); Broward Health Medical Center, Fort Lauderdale, FL (Dr Hector Rodriguez-Cortes); C S Mott Children's Hospital, Ann Arbor, MI (Dr Rajen Mody); Cardinal Glennon Children's Medical Center, St Louis, MO (Dr William Ferguson); Cardon Children's Medical Center, Mesa, AZ (Dr Xiaxin Li); Carilion Clinic Children's Hospital, Roanoke, VA (Dr Mandy Atkinson); Carolinas Medical Center/Levine Cancer Institute, Charlotte, NC (Dr Joel Kaplan); Cedars-Sinai Medical Center, Los Angeles, CA (Dr Fataneh (Fae) Majlessipour); Children's Healthcare of Atlanta - Egleston, Atlanta, GA (Dr Howard Katzenstein); Children's Hospital and Medical Center of Omaha, Omaha, NE (Dr Minnie Abromowitch); Children's Hospital and Research Center at Oakland, Oakland, CA (Dr Carla Golden); Children's Hospital Central California, Madera, CA (Dr Vonda Crouse); Children's Hospital Colorado, Aurora, CO (Dr Kelly Maloney); Children's Hospital Medical Center of Akron, Akron, OH (Dr Steven Kuerbitz); Children's Hospital of Alabama, Birmingham, AL (Dr Alyssa Reddy); Children's Hospital of Los Angeles, Los Angeles, CA (Dr Leo Mascarenhas); Children's Hospital of Philadelphia, Philadelphia, PA (Dr Frank Balis); Children's Hospital of Pittsburgh of UPMC, Pittsburgh, PA (Dr Arthur Ritchey); Children's Hospital-Main Campus, New Orleans, LA (Dr Lolie Yu); Children's Hospitals and Clinics of Minnesota - Minneapolis, Minneapolis, MN (Dr Bruce Bostrom); Children's Mercy Hospitals and Clinics, Kansas City, MO (Dr Maxine Hetherington); Children's National Medical Center, Washington, DC (Dr Jeffrey Dome); Childrens Hospital of Orange County, Orange, CA (Dr Violet Shen); Childrens Hospital-King's Daughters, Norfolk, VA (Dr Eric Lowe); Cincinnati Children's Hospital Medical Center, Cincinatti, OH (Dr John Perentesis); City of Hope, Duarte, CA (Dr Anna Pawlowska); Cleveland Clinic Foundation, Cleveland, OH (Dr Margaret Thompson); Columbia University Medical Center, New York, NY (Dr Alice Lee); Connecticut Children's Medical Center, Hartford, CT (Dr Michael Isakoff); Cook Children's Medical Center, Fort Worth, TX (Dr Mary Meaghan Granger); Midwest Children's Cancer Center, Milwaukee, WI (Dr Michael Kelly); Miller Children's Hospital, Long Beach, CA (Dr Amanda Termuhlen); Mission Hospitals Inc, Asheville, NC (Dr Orren Beaty); Montefiore Medical Center - Moses Campus, Bronx, NY (Dr Rosanna Ricafort); Morristown Medical Center, Morristown, NJ (Dr Steven Halpern); Mount Sinai Medical Center, New York, NY (Dr Birte Wistinghausen); Natalie W Bryant Cancer Center, Tulsa, OK (Dr Gregory Kirkpatrick); Nationwide Children's Hospital, Colombus, OH (Dr Mark Ranalli); Naval Medical Center - Portsmouth, Fort Sam Houston, TX (Dr Della Howell); Naval Medical Center -San Diego, Fort Sam Houston, TX (Dr Della Howell); Nemours Children's Clinic - Jacksonville, Jacksonville, FL (Dr Eric Sandler); Nemours Children's Clinic - Pensacola, Pensacola, FL (Dr Jeffrey Schwartz); Nemours Childrens Clinic - Orlando, Orlando, FL (Dr Ramamoorthy Nagasubramanian); Nevada Cancer Research Foundation CCOP, Las Vegas, NV (Dr Jonathan Bernstein); New York Medical College, Valhalla, NY (Dr Mehmet Ozkaynak); Newark Beth Israel Medical Center, Newark, NJ (Dr Peri Kamalakar); Ochsner Clinic Foundation, New Orleans, LA (Dr Rajasekharan (Raj) Warrier); Oregon Health and Science University, Portland, OR (Dr Linda Stork); Palmetto Health Richland, Columbia, SC (Dr Ronnie Neuberg); Penn State Hershey Children's Hospital, Hershey, PA (Dr Lisa McGregor); Phoenix Childrens Hospital, Phoenix, AZ (Dr Jessica Boklan); Novant Health Presbyterian Medical Center, Charlotte, NC (Dr Paulette Bryant); Primary Children's Medical Center, Salt Lake City, UT (Dr Phillip Barnette); Providence Sacred Heart Medical Center and Children's Hospital, Spokane, WA (Dr Judy Felgenhauer); Rady Children's Hospital - San Diego, San Diego, CA (Dr William Roberts); Rainbow Babies and Childrens Hospital, Cleveland, OH (Dr Yousif (Joe) Matloub); Randall Children's Hospital at Legacy Emanuel, Portland, OR (Dr Janice Olson); Raymond Blank Children's Hospital, Des Moines, IA (Dr Wendy Woods-Swafford); Rhode Island Hospital, Providence, RI (Dr Kevin Schwartz); Riley Hospital for Children, Indianapolis, IN (Dr Robert Fallon); Rocky Mountain Hospital for Children-Presbyterian Saint Luke's Medical, Denver, CO (Dr Jennifer Clark); Roswell Park Cancer Institute, Buffalo, NY (Dr Martin Brecher); Saint Barnabas Medical Center (Newark Beth Israel Medical Center), Livingston, NJ (Dr Stacey Rifkin-Zenenberg); Saint Christopher's Hospital for Children, Philadelphia, PA (Dr Gregory Halligan); Saint John Hospital and Medical Center, Gross Pointe Woods, MI (Dr Hadi Sawaf); Saint Joseph Children's Hospital of Tampa, Tampa, FL (Dr Hardeo Panchoosingh); Saint Joseph's Regional Medical Center, Peterson, NJ (Dr Mary Bonilla); Saint Jude Children's Research Hospital, Memphis, TN (Dr Wayne Furman); Saint Jude Midwest Affiliate, Peoria, IL (Dr Pedro De Alarcon); Saint Luke's Mountain States Tumor Institute, Boise, ID (Dr Eugenia Chang); Saint Mary's Hospital, West Palm Beach, FL (Dr Narayana Gowda); Saint Peter's University Hospital, New Brunswick, NJ (Dr Stanley Calderwood); Saint Vincent Hospital, Green Bay, WI (Dr John Hill); Saint Vincent Hospital and Health Services, Indianapolis, IN (Dr Bassem Razzouk); San Jorge Children's Hospital, Santurce, PR (Dr Luis Clavell); Sanford Medical Center-Fargo, Fargo, ND (Dr Nathan Kobrinsky); Sanford USD Medical Center - Sioux Falls, Sioux Falls, ND (Dr Kayelyn Wagner); Santa Barbara Cottage Hospital, Santa Barbara, CA (Dr Daniel Greenfield); Scott and White Memorial Hospital, Temple, TX (Dr Guy Grayson); Seattle Children's Hospital, Seattle, WA (Dr Douglas Hawkins); Sinai Hospital of Baltimore, Baltimore, MD (Dr Joseph Wiley); Southern California Permanente Medical Group, Los Angeles, CA (Dr Robert Cooper); Southern Illinois University School of Medicine, Springfield, IL (Dr Gregory Brandt); State University of New York Upstate Medical University, Syracuse, NY (Dr Karol Kerr); Stony Brook University Medical Center, Stony Brook, NY (Dr Robert Parker); T C Thompson Children's Hospital, Chattanooga, TN (Dr Manoo Bhakta); Tampa General Hospital, Tampa, FL (Dr Cameron Tebbi); Texas Tech University Health Science Center-Amarillo (Children' Hospital of San Antonio, San Antonio, TX (Dr Osvaldo Regueira); The Children's Medical Center of Dayton (Dayton Children's Hospital), Dayton, OH (Dr Emmett Broxson); The Steven and Alexandra Cohen Children's Medical Center of New York, New Hyde Park, NY (Dr Arlene Redner); The Toledo Hospital/Toledo Children's Hospital, Toledo, OH (Dr Dagmar Stein); The University of Arizona Medical Center-University Campus, Tuscon, AZ (Dr Brenda Wittman); Tripler Army Medical Center, Fort Sam Houston, TX (Dr Della Howell); UMDNJ - Robert Wood Johnson University Hospital (Rutgers Cancer Institute of New Jersey), New Brunswick, NJ (Dr Richard Drachtman); University of Arkansas for Medical Sciences (Arkansas Children's Hospital), Little Rock, AK (Dr David Becton); University of California Davis-Cancer Center, Sacramento, CA (Dr Jay Michael Balagtas); University of California San Francisco Medical Center-Parnassus, San Francisco, CA (Dr Robert Goldsby); University of Chicago, Chicago, IL (Dr Susan Cohn); University of Florida, Gainesville, FL (Dr William Slayton); University of Hawaii, Honolulu, HI (Dr Robert Wilkinson); University of Illinois, Chicago, IL (Dr Mary Schmidt); University of Iowa Hospitals and Clinics, Iowa City, IA (Dr Ayman El-Sheikh); University of Kentucky, Lexington, KY (Dr Martha Greenwood); University of Maryland Greenebaum Cancer Center, Baltimore, ND (Dr Teresa York); University of Massachusetts Medical School, Worcester, MA (Dr Christopher Keuker); University of Miami Miller School of Medicine-Sylvester Cancer Center, Miami, FL (Dr Julio Barredo); University of Minnesota Medical Center-Fairview, Minneapolis, MN (Dr Joseph Neglia); University of Mississippi Medical Center, Jackson, MS (Dr Gail Megason); University of Missouri-Columbia (MUHC-Women and Children's Hospital), Columbia, MO (Dr Thomas Loew); University of Nebraska Medical Center, Omaha, NE (Dr Peter Coccia); University of New Mexico, Albuquerque, NM (Dr Koh Boayue); University of North Carolina at Chapel Hill, Chapel Hill, NC (Dr Stuart Gold); University of Oklahoma Health Sciences Center, Oklahoma City OK (Dr Rene McNall-Knapp); University of Rochester, Rochester, NY (Dr Jeffrey Andolina); University of South Alabama, Mobile, AL (Dr Felicia Wilson); University of Texas Health Science Center, San Antonio, TX (Dr Anne-Marie Langevin); University of Texas Southwestern Medical Center, Dallas, TX (Dr Naomi Winick); University of Vermont, Burlington, VT (Dr Alan Homans); University of Virginia, Charlottesville, VA (Dr Kimberly Dunsmore); University of Wisconsin Hospital and Clinics, Madison, WI (Dr Kenneth De Santes); Vanderbilt University, Nashville, TN (Dr Haydar Frangoul); Wake Forest University Health Sciences, Winston-Salem, NC (Dr Thomas McLean); Walter Reed National Military Medical Center, Fort Sam Houston, TX (Dr Della Howell); Washington University School of Medicine, St Louis, MO (Dr Robert Hayashi); Wayne State University, Detroit, MI (Dr Zhihong Wang); Weill Medical College of Cornell University, New York, NY (Dr Alexander Aledo); William Beaumont Hospital (Beaumont Children's Hospital-Royal Oak), Royal Oak, MI (Dr Charles Main); Winthrop University Hospital, Mineola, NY (Dr Mark Weinblatt); Yale University, New Haven, CT (Dr Nina Kadan-Lottick); Covenant Children's Hospital, Lubbock, TX (Dr Latha Prasannan); Dana-Farber Cancer Institute, Boston, MA (Dr Carlos Rodriguez-Galindo); Dartmouth Hitchcock Medical Center, Lebanon, NH (Dr Sara Chaffee); Dell Children's Medical Center of Central Texas, Austin, TX (Dr Sharon Lockhart); Driscoll Children's Hospital, Corpus Christie, TX (Dr M. Cris Johnson); Duke University Medical Center, Durham, NC (Dr Susan Kreissman); East Carolina University, Greeneville, NC (Dr Mauro Grossi); East Tennessee Childrens Hospital, Knoxville, TN (Dr Ray Pais); Eastern Maine Medical Center, Brewer, ME (Dr Sarah Fryberger); Georgia Regents University, Augusta, GA (Dr Colleen McDonough); Hackensack University Medical Center, Hackensack, NJ (Dr Burton Appel); Helen DeVos Children's Hospital at Spectrum Health, Grand Rapids, MI (Dr David Dickens); Hurley Medical Center, Flint, MI (Dr Susumu Inoue); Inova Fairfax Hospital, Falls Church, VA (Dr Marshall Schorin); Johns Hopkins University, Baltimore, MD (Dr Kenneth Cohen); Kaiser Permanente-Oakland, Oakland, CA (Dr Steven Bergstrom); Kosair Children's Hospital, Louisville, KY (Dr Salvatore Bertolone); Lehigh Valley Hospital - Muhlenberg, Bethlehem, PA (Dr Philip Monteleone); Loma Linda University Medical Center, Loma Linda, CA (Dr Antranik Bedros); Loyola University Medical Center, Maywood, IL (Dr Ricarchito Manera); Lucile Packard Children's Hospital Stanford University, Palo Alto, CA (Dr Neyssa Marina); M D Anderson Cancer Center, Houston, TX (Dr Najat Daw); Madigan Army Medical Center, Fort Sam Houston, TX (Dr Della Howell); Maine Children's Cancer Program, Scarsborough, ME (Dr Eric Larsen); Mark O Hatfield-Warren Grant Magnuson Clinical Center, Bethesda, MD (Dr Crystal Mackall); Marshfield Clinic, Marshfield, WI (Dr Michael McManus); Mary Bridge Children's Hospital and Health Center, Tacoma, WA (Dr Robert Irwin); Massachusetts General Hospital Cancer Center, Boston, MA (Dr Howard Weinstein); Mattel Children's Hospital UCLA, Los Angeles, CA (Dr Pamela Kempert); Mayo Clinic, Rochester, MN (Dr Carola Arndt); Medical City Dallas Hospital, Dallas, TX (Dr Carl Lenarsky); Medical University of South Carolina, Charleston, SC (Dr Jacqueline Kraveka); MedStar Georgetown University Hospital, Washington, DC (Dr Aziza Shad); Memorial Regional Hospital/Joe DiMaggio Children's Hospital, Hollywood, FL (Dr Iftikhar Hanif); Memorial Sloan Kettering Cancer Center, New York, NY (Dr Peter Steinherz); Memorial University Medical Center, Savannah, GA (Dr J. Martin Johnston); Mercy Children's Hospital, Toledo, OH (Dr Rama Jasty); Mercy Hospital Saint Louis, St Louis, MO (Dr Robin Hanson); Methodist Children's Hospital of South Texas, San Antonio, TX (Dr Jaime Estrada); Miami Children's Hospital, Miami, FL (Dr Enrique Escalon); Michigan State University Clinical Center, East Lansing, MI (Dr Renuka Gera); New York University Langone Medical Center, (Dr Elizabeth Raetz).

#### Canada.

Alberta Children's Hospital, Calgary, AB (Dr Douglas Strother); Allan Blair Cancer Centre, Regina, SK (Dr Mansoor Haq); British Columbia Children's Hospital, Vancouver, BC (Dr Caron Strahlendorf); Cancer Centre of Southeastern Ontario at Kingston General Hospital, Kingston, ON (Dr Mariana Silva); CancerCare Manitoba, Winnipeg, MB (Dr Rochelle Yanofsky); Centre Hospitalier Universitaire Sainte-Justine, Montreal, QC (Dr Yvan Samson); Children's Hospital, London, ON (Dr Anne Cairney); Children's Hospital of Eastern Ontario, Ottawa, ON (Dr Jacqueline Halton); CHU de Quebec, Quebec, PQ (Dr Bruno Michon); Saskatoon Cancer Centre, Saskatoon, SK (Dr Christopher Mpofu); The Montreal Children's Hospital of the MUHC, Montreal, QC (Dr Sharon Abish); University of Alberta Hospital, Edmonton, AB (Dr Sunil Jayantilal Desai); Hospital for Sick Children, Toronto, ON (Dr Ronald Grant); IWK Health Centre, Halifax, NS (Dr Margaret Yhap); Janeway Child Health Centre, St John's, NF (Dr Lisa Anne Goodyear); McMaster Children's Hospital at Hamilton Health Sciences, Hamilton, ON (Dr Carol Portwine).

#### Australia.

Monash Medical Center-Clayton Campus, Clayton, VIC (Dr Peter Downie); Princess Margaret Hospital for Children, Perth, WA. (Dr Catherine Cole); Royal Children's Hospital, Parkville, VIC (Dr Francoise Mechinaud); Royal Childrens Hospital-Brisbane, Brisbane, QLD (Dr Helen Irving); Sydney Children's Hospital, Randwick, NSW (Dr Draga Barbaric); The Children's Hospital at Westmead, Westmead, NSW (Dr Geoffrey McCowage); Women's and Children's Hospital-Adelaide, North Adelaide, SA (Dr Maria Kirby).

#### Switzerland.

Swiss Pediatric Oncology Group - Bern, Bern (Dr Roland Ammann); Swiss Pediatric Oncology Group - Geneva, Geneva (Dr Marc Ansari); Swiss Pediatric Oncology Group - Lausanne, Lausanne (Dr Maja Beck Popovic).

#### New Zealand.

Christchurch Hospital, Christchurch (Dr Michael Sullivan); Starship Children's Hospital, Auckland (Dr Teague).

#### Mexico.

Hospital Infantil de Mexico Federico Gomez, Mexico City (Dr Aurora Medina-Sanson).

### COG Panel Representatives and Additional Contributors

Neyssa Marina, Chief Investigator; TMG Member: Chief Investigator; National Coordinator; Oncology Panel

Mark Bernstein, Chief Investigator; TMG Chair; National Coordinator; Oncology Panel

Caroline Wang, TMG Member: Research Coordinator

Justin Davis, TMG Member: Research Coordinator

Possidonia Gontijo, TMG Member: Research Coordinator

Mark Krailo, TMG Member: Statistician

**Statistics Panel:**

Allen Goorin, Oncology Panel

Paul Meyers, Oncology Panel

Lisa Teot,* Pathology Review Panel

Richard Gorlick,* Biological Studies Panel

Chand Khanna, Biological Studies Panel

Ching Lau, Biological Studies Panel

Doug Letson,* Surgical Panel

Mark Gebhardt, Surgical Panel

Lor Randall, Surgical Panel

James Meyer, Radiology Panel

Helen Nadel, Radiology Panel

Paula Schomberg, Radiotherapy Panel

Amy Barr, Pharmacy Panel

(*) Group representative

### A2. Cooperative Osteosarkom Studiengruppe (COSS)

#### Austria.

(Univers) Kinderklinik, Graz (Herrn Univ. Prof. Dr C. Urban); Univers. Kinderklinik, Innsbruck (Herrn Prof. Dr B. Meister); LKA Salzburg, Salzburg (Herrn OA Dr N. Jones).

#### Czech Republic.

Univ. Childrens Hosp. Brno, Brno (Herrn Prof. Dr P. Mudry); University Hospital MOTOL, Prag (Herrn Dr med. H. Mottl).

#### Germany.

Universitätskinderklinik, Aachen (Herrn Prof. Dr R. Mertens); Krh. -Zweckverband Augsburg, Augsburg (Frau OÄ Dr A. K. Gnekow); Zentralklinikum, Augsburg (Herrn Prof. Dr G. Schlimok); Helios Kliniken Bad Saarow, Bad Saarow (Herrn PD Dr med. Reichardt); Sozialstiftung Bamberg, Klinikum am Bruderwald, Bamberg (Herrn Priv.-Doz. Dr med. Roland Repp); Klinikum Bayreuth, Bayreuth (Herrn Prof. Dr Th. Rupprecht); Charité Campus Virchow Klinikum Berlin, Berlin (Frau Prof. Dr med. A. Eggert); Ev. Waldkrankenhaus Spandau, Berlin (Herrn Dr Potenberg); Ev. Waldkrankenhaus Spandau, Berlin (Herrn Prof. Dr Noack); Helios Klinikum Berlin - Buch, Berlin (Herrn PD Dr med. Reichardt); Helios Klinikum Berlin-Buch; Klinik für Kinder und Jugendmedizin, Berlin (Herrn Prof. Dr L. Schweigerer); Charite, Berlin (Herrn Dr Jan Eucker); Charite - Campus Virchow Klinikum, Berlin (Herrn OA Dr P. Thuss-Patience); Ev. Krankenhaus Bielefeld gGmbH, Bielefeld (Herrn OA Dr Jorch); Ev. Krankenhaus, Bielefeld (Herrn PD Dr med. F. Weißinger); Univers. Kinderklinik, Bonn (Herrn Dr med. Stefan Schönberger); Städt. Klinikum Holwedestraße, Braunschweig (Herrn OA Dr med. Eberl); Klinikum Bremen - Mitte gGmbh / Prof.-Hess-Kinderklinik, Bremen (Herrn Prof. Dr Pekrun); Klinikum Bremen-Mitte, Bremen (Frau Dr med. Henrike Thomssen); Ev. Diakonie-Krankenhaus, Bremen (Herrn Prof. Dr Pflüger); Klinikum Chemnitz, Chemnitz (Herrn OA Dr med. A. Hofmann); Klinikum Chemnitz gGmbH, Chemnitz (Herrn PD Dr med. M. Hänel); Kliniken Dortmund, Dortmund (Frau Dr S. Bäumer); Städt. Kliniken, Dortmund (Herrn Prof. Dr med. Schneider); Univers. Kinderklinik, Dresden (Herrn Prof. Dr med. M. Suttorp); Med. Universitätsklinik I, Dresden (Herrn PD Dr G. Folprecht); Univers. Kinderklinik, Düsseldorf (Herrn Prof. Dr Borkhardt); Med. Universitätsklinik & Poliklinik, Düsseldorf (Herrn Prof. Dr R. Haas); Helios Klinikum Erfurt, Erfurt (Herrn CA PD Dr Sauerbrey); Universitätsklinikum Erlangen Kinder- und Jugendklinik, Erlangen (Herrn PD Dr med. Markus Metzler); Universitätsklinikum Erlangen, Erlangen (Herrn PD Dr med. Spriewald); Waldkrankenhaus St. Marien, Erlangen (Herrn PD Dr med. G. Preclik); Univers. Kinderklinik, Essen (Prof. Dr Bernhard Kremens); Univers. Kinderklinik, Frankfurt (Herrn Prof. Dr Klingebiel); Universitätsklinik, Frankfurt (Herrn Prof. Dr Bergmann); Universitätsklinikum Freiburg Zentrum für Kinder- und Jugendmedizin, Freiburg (Frau Prof. Dr med. Charlotte Niemeyer); Klinik Sanafontis, Freiburg (Herrn Dr med. J. Rentschler); Klinik Sanafontis, Freiburg (Herrn PD Dr med. J. Drevs); Univers. Kinderklinik, Gießen (Herrn Prof. Dr Reiter); Univ. Klinikum Gießen, Gießen (Herrn Prof. Dr med. M. Rummel); Universitätskinderklinik Göttingen, Göttingen (Herrn Prof. Dr med. Lakomek); Medizinische Klinik & Poliklinik, Göttingen (Herrn Prof. Dr L. Trümper); Univ. Klinikum Greifswald, Greifswald (Herrn Prof. Dr med. H. Lode); Universitätsmedizin Greifswald, Greifswald (Herrn Dr Christian-Andreas Schmidt); Krankenhaus St. Elisabeth / St. Barbara, Halle (Frau OÄ Dr G. Günther); Med. Universitätsklinik / Innere IV, Halle (Herrn Dr med. H.-H.- Wolf); Universitätsklinik f. Kinder & Jugendmedizin, Halle / Saale (Herrn Prof. Dr Körholz); Univers. Kinderklinik, Hamburg (Herrn Dr med. Hassenpflug); Med. Hochschule Hannover, Hannover (Herrn Prof. Dr A. Beilken); MHH Zentrum Innere Medizin, Hannover (Herrn Prof. Dr med. Christoph Reuter); Klinikum Siloah, Hannover (Herrn PD Dr Kirchner); Univers. Klinik f. Kinder & Jugendmedizin, Heidelberg (Herrn OA Dr med. Wolfgang Behnisch); Med. Klinik & Poliklinik V, Heidelberg (Frau OÄ Prof. Dr Egerer); Orthop. Universitätsklinik I, Heidelberg (Herrn Prof. Dr Ewerbeck); SLK Kliniken Heilbronn, Heilbronn (Frau Dr M. Streiter); Gemeinschaftskrankenhaus Herdecke, Herdecke (Herrn Dr med. A. Längler); Univers. Kinderklinik, Homburg (Herrn Prof. Dr Graf); Med. Universitätsklinik I, Homburg (Herrn Prof. Dr Pfreundschuh); Univers. Kinderklinik, Jena (Herrn OA. Dr med. Gruhn); Universitätsklinikum Jena, Jena (Herrn Dr med. Schmalenberg); Städt. Klinikum Karlsruhe, Karlsruhe (Herrn Dr med. A. Leipold); Klinikum Kassel, Kassel (Frau PD Dr med. Nathrath); Universitätsklinikum Schleswig - Holstein, Kiel (Herrn Prof. Dr M. Schrappe); Universitätsklinikum Schleswig-Holstein, Kiel (Herrn Prof. Dr Kneba); Gemeinschaftsklinikum Kemperhof Koblenz, Koblenz (Herrn OA Dr Ferrari); Uniklinik Köln, Köln (Herrn Prof. Dr med. T. Simon); Med. Universitätsklinik I, Köln (Herrn Prof. J-M. Chemnitz); Helioskliniken Krefeld, Krefeld (Herrn Thomas Imschweiler); Univers. Kinderklinik Leipzig, Leipzig (Herrn Prof. Dr med. Christiansen); Universitätsklinikum Leipzig, Leipzig (Herrn Prof. Dr med. Dr h. c. Niederwieser); Landes- Frauen- und Kinderklinik Linz, Linz (Herrn Prof. Dr K. Schmitt); Univers. Kinderklinik, Lübeck (Herrn PD Dr med. M. Lauten); Klinikum Lüdenscheid, Lüdenscheid (Herrn Prof. Dr med. G. Heil); Klinikum d. Stadt Ludwigshafen, Ludwigshafen (Herrn Prof. Dr Uppenkamp); St. Marien u. St. Annastiftskrankenhaus Ludwigshafen/Rh, Ludwigshafen (Abteilung aufgelöst); Universitätsklinikum Magdeburg A.ö.R Universitätskinderklinik, Magdeburg (Herrn Prof. Dr med. Vorwerk); Med. Universitätsklinik III, Mainz (Herrn OA Dr H. J. Beck); Univers. Kinderklinik Mainz, Mainz (Herrn PD Dr Jörg Faber); Universitätskinderklinik Mannheim, Mannheim (Herrn PD Dr Dürken); Univers. Kinderklinik, Marburg (Frau Dr med. Barbara Schütz); Johannes Wesling Klinikum Minden, Minden (Herrn PD Dr Erdlenbruch); Kinderklinik & Poliklinik d. TU München, München (Frau PD Dr med. Irene Teichert von Lüttichau); III. Medizinische Klinik & Poliklinik re. d. Isar, München (Herrn PD Dr I. Ringshausen); Dr von Haunersches Kinderspital, München (Frau OÄ PD Dr med. I. Schmid); Klinikum Großhadern, München (Herrn PD Dr Lars Lindner); Medizinische Klinik & Poliklinik, Münster (Herrn Prof. Dr Berdel); Universitätsklinikum Münster, Münster (Herrn Prof. Dr Jürgens); Cnopf´sche Kinderklinik, Nürnberg (Herrn Prof Dr Scheurlen); Med. Universitätsklinik V, Nürnberg (Herrn Prof. Dr M. Wilhelm); Pius-Hospital, Oldenburg (Herrn Prof. Dr F. Griesinger); Klinikum Oldenburg gGmbH, Oldenburg (Herr Prof. Dr Müller); Brüderkrankenhaus St. Josef Paderborn, Paderborn (Herrn CA Dr med. T. Gaska); Klinikum Passau, Passau (Herrn CA PD Dr T. Südhoff); Krhs. Barmherzige Brüder, Regensburg (Herrn Prof. Dr med. Jan Braess); Med. Universitätsklinik, Regensburg (Frau Dr med. Ch. Hart); Univ. Kinderklinik Regensburg, Regensburg (Herrn Prof. Dr med. Corbacioglu); Universitätsklinikum Rostock, Rostock (Herrn PD Dr med. C. F. Classen); Diakoniekrankenhaus Rotenburg, Rotenburg (Herrn Dr med. F. Heits); Klinikum Saarbrücken, Saarbrücken (Herrn Dr Joachim Weis); Diakonie - Klinikum, Schwäbisch Hall (Herrn Dr Thomas Geer); Helios Kliniken Schwerin, Schwerin (Herrn OA Dr med. Kasbohm); Heliosklinikum Schwerin, Schwerin (Herrn Dr D. Hähling); Asklepios Klinik St. Augustin, St. Augustin (Herrn Dr med. Harald Reinhard); Klinikum Stuttgart; Zentrum für Kinder- und Jugendmedizin - Olgahospital, Stuttgart (Herrn Prof. Dr Bielack); Diakonie-Klinikum-Stuttgart, Stuttgart (Frau Prof. Dr Heidemann); Marienhospital, Stuttgart (Herrn Prof. Dr med. Denzlinger); Klinikum Stuttgart/Krh. Bad Cannstatt, Stuttgart (Herrn Prof. Dr med. Andus); Univers. Kinderklinik, Tübingen (Herrn Prof. Dr med. Rupert Handgretinger); Univers. Kinderklinik & Poliklinik, Ulm (Herrn Prof. Dr Debatin).

#### Hungary.

Állami Egészségügyi Központ, Budapest (Frau Dr Zsuzsanna Pápai); SE Orthopädische Klinik, Budapest (Herrn Prof. Dr M. Szendroi); II. Kinderklinik der Semmelweis Universität, Budapest (Herrn Dr Gabor Kovacs).

#### Switzerland.

Kantonspital Aarau, Aarau (Frau OÄ Dr Angst); Universitäts-Kinderspital beider Basel, Basel (Herrn Prof. Dr med. Thomas Kühne); Ospedale San Giovanni, Bellinzona (Herrn Dr P. Brazzola); Kantonsspital Luzern, Luzern 16 (Herrn PD Dr Rischewski).

### COSS Panel Representatives and Additional Contributors

Stefan Bielack, Chief Investigator; TMG Member: Chief Investigator; National Coordinator: Germany, Oncology Panel: Paediatric Oncology

Beate Kempf-Bielack, TMG Member: Study Physician

Miriam Wilhelm, TMG Member: Study Physician

Katja Zils, TMG Member: Study Physician

Joachim Gerss, TMG Member: Statistician

Matthias Kevric, TMG Member: Data Manager

Benjamin Sorg, TMG Member: Data Manager

Dorothe Carrle, TMG Member: Study Physician

Feven Assefaw: Secretary

Lynn Hazlewood: Secretary

Claudia Wurster: Secretary

Leo Kager, National Coordinator: Austria; Oncology Panel: Paediatric Oncology

Zsuzsanna Pápai, National Coordinator: Hungary

Thomas Kühne, National Coordinator: Switzerland

Hubert Mottl, National Coordinator: Czech Republic

Thomas Kühne, Oncology Panel: Paediatric Oncology

Peter Reichardt, Oncology Panel: Medical Oncology

Zsuzsanna Pápai, Oncology Panel: Medical Oncology

Udo Kontnyl, Oncology Panel: Paediatric Oncology

W. Hassenpflug, Oncology Panel: Paediatric Oncology

Norbert Graf, Oncology Panel: Paediatric Oncology

Michaela Nathrath, Oncology Panel: Paediatric Oncology; Biological Studies Panel

Wolfgang E. Berdel, Oncology Panel: Medical oncology

Heribert Jürgens, Oncology Panel: Paediatric Oncology

Regine Meyer-Steinacker, Oncology Panel: Medical Oncology

Gernot Jundt,* Pathology Review Panel

Andreas Schulz, Pathology Review Panel

Günter Delling, Pathology Review Panel

Mathias Werner, Pathology Review Panel

Albert Roessner, Pathology Review Panel

Gabriele Köhler (*associate*: Ch. Dierkes), Pathology Review Panel

Horst Bürger, Pathology Review Panel

Susanna Lang (*associate*: Irene Sulzbacher), Pathology Review Panel

Arthur R. von Hochstetter, Pathology Review Panel

Gabriela Arato, Pathology Review Panel

Gernot Jundt,* Biological Studies Panel

W. Hassenpflug, Biological Studies Panel

Rainhard Windhager* (*associate*: Martin Dominkus), Surgical Panel

Per-Ulf Tunn, Surgical Panel

Miklos Szendroi (*associate*:. Imre Antal), Surgical Panel

Detlev Branscheid, Surgical Panel: Thoracic Surgery Representative

Godehard Friedel, Surgical Panel: Thoracic Surgery Representative

Ulrich Heise, Surgical Panel

Volker Ewerbeck, Surgical Panel

H. Roland Dürr, Surgical Panel

Axel Hillmann, Surgical Panel

Georg Gosheger (*associate*: Jendrik Hardes), Surgical Panel

G. Ulrich Exner, Surgical Panel

Joachim Sciuk, Radiology Panel: Nuclear Medicine

Knut Helmke, Radiology Panel: Diagnostic Radiology

Peter Winkler (*associate: Thekla v. Kalle*), Radiology Panel: Diagnostic Radiology

Reiner Maas, Radiology Panel: Diagnostic Radiology

Christiane Franzius, Radiology Panel: Nuclear Medicine

Joachim Gerss, Statistics Panel

Rudolf Schwarz, Radiotherapy Panel

Beate Timmermann, Radiotherapy Panel: Particle Therapy

Elvira Ahlke & Hedwig Kolve, Pharmacy Panel

(*) Group representative

### A3. European Osteosarcoma Intergroup (EOI)

#### United Kingdom.

University College Hospital, London (Prof. Jeremy Whelan); Royal Hospital for Sick Children, Edinburgh (Dr Hamish Wallace); Birmingham Childrens Hospital, Birmingham (Dr Bruce Morland); Sheffield Children's Hospital, Sheffield (Dr Vicki Lee, Dr Mary Gerrard); Christie Hospital, Manchester (Dr Bernadette Brennan); Royal Manchester Children's Hospital, Manchester (Dr Bernadette Brennan); Addenbrooke's Hospital, Cambridge (Dr Amos Burke); Royal Marsden Hospital, London (Dr Julia Chisholm, Prof. Kathy Pritchard-Jones); Royal Hospital for Sick Children, Glasgow (Dr Milind Ronghe); Beatson West of Scotland Cancer Centre, Glasgow (Dr Jeff White); Royal Aberdeen Childrens Hospital, Aberdeen (Dr Derek King); Queens Medical Centre, Nottingham (Prof. David Walker); St James University Hospital, Leeds (Dr Susan Picton, Dr Ian Lewis); Children's Hospital for Wales, Cardiff (Dr Heidi Traunecker); Royal Orthopaedic Hospital, Birmingham (Dr David Peake); Royal Victoria Infirmary, Newcastle (Dr Juliet Hale); Nottingham University Hospitals, City Campus, Nottingham (Dr Ivo Hennig, Dr Michael Sokal); Southampton General Hospital, Southampton (Dr Gary Nicolin); Bristol Royal Hospital for Children, Bristol (Prof. Mike Stevens); Ninewells Hospital, Dundee (Dr Michelle Ferguson, Prof. Elaine Rankin); Weston Park Hospital, Sheffield (Prof. Penella Woll); Leicester Royal Infirmary, Leicester (Dr Johannes Visser); Clatterbridge Centre for Oncology, Liverpool (Dr Peter Clark, Dr Nasim Ali); Churchill Hospital, Oxford (Prof. Bass Hassan); Royal Liverpool Childrens Hospital, Liverpool (Dr Barry Pizer, Dr Heather McDowell); Bristol Haematology & Oncology Centre, Bristol (Prof. Mike Stevens); Newcastle General Hospital, Newcastle (Dr Mark Verrill).

#### Netherlands.

Leiden University Medical Center, Leiden (Prof. Hans Gelderblom, Dr Jacob Anninga); Emma's Children's Hospital/AMC Amsterdam, Amsterdam (Dr Henk Van Den Berg); Radboud University Nijmegen Medical Centre, Nijmegan (Prof. Hoogerbrugge); University Medical Center Groningen, Groningen (Prof. WA Kamps, Dr ESJM De Bont).

#### Belgium.

University Hospital Ghent, Ghent (Dr Catharina Dhooge); UZ Brussels, Brussels (Dr An Van Damme, Dr Jutte Van der Werff ten Bosch); Jules Bordet Institute, Brussels (Dr Thierry Gil); Universitaire Ziekenhuizen Leuven, Leuven (Dr Marleen Renard); Cliniques Universitaires Saint-Luc, Brussels (Prof. Benedicte Brichard); Hopital Universitaire des Enfants, Brussels (Dr Sophie Huybrechts).

#### Ireland.

Our Lady's Children's Hospital, Dublin (Dr Michael Capra).

### EOI Panel Representatives and Additional Contributors

Jeremy Whelan, Chief Investigator; National Coordinator: United Kingdom; Oncology Panel ; TMG Member: Chief Investigator

Nicola Joffe, TMG Member; Clinical Project Manager

Sue Fleck, TMG Member; Clinical Project Manager

Karen Sanders, TMG Member; Clinical Project Manager; Data Manager

Barbara Uscinska, TMG Member; Clinical Project Manager

James Pickering, TMG Member; Trial Manager; Data Manager

Gintare Rutkauskaite. TMG Member; Trial Manager

Aurelie Faysse, TMG Member; Trial Manager

Sarah Beall, TMG Member; Trial Manager

Beth May, TMG Member; Trial Manager

Anne Holliday, TMG Member; Trial Manager

Monica Tomiczek, TMG Member; Trial Manager, Data Manager

Matthew Sydes, TMG Member; Trial Statistician; Statistics Panel

Gordana Jovic, TMG Member; Statistician

Jane Hook, TMG Member; Study Physician

Leonardo Trani, TMG Member; Study Physician

Martha Perisoglou, TMG Member; Study Physician

Catharina Dhooge (Claeys), National Coordinator: Belgium

Rachel Jinks, TMG Member; Statistician

**Oncology Panel:**

Michael Capra, National Coordinator: Ireland

Jakob Anninga, National Coordinator: Netherlands, Oncology Panel

Hans Gelderblom, National Coordinator: Netherlands

Bruce Morland, Oncology Panel

Ian Lewis, Oncology Panel

Pancras Hogendoorn,* Pathology Review Panel; Biological Studies Panel

Judith V.M.G. Bovee, Pathology Review Panel

J. Bras, Pathology Review Panel

Adrienne Flanagan, Pathology Review Panel

Elaine MacDuff, Pathology Review Panel

Robin Reid, Pathology Review Panel

Petra Dildey, Pathology Review Panel

David E Hughes, Pathology Review Panel

Esther Hauben, Pathology Review Panel

Chas Mangham, Pathology Review Panel

Anne Marie Cleton Jansen, Biological Studies Panel

Rob Grimer,* Surgical Panel

Steve Cannon, Surgical Panel

Anthony Taminiau, Surgical Panel

Michael Gebhart, Surgical Panel

Mark Davies, Radiology Panel

Paul O'Donnell, Radiology Panel

William Ramsden, Radiology Panel

H.J. van der Woude, Radiology Panel

Koenraad Verstraete, Radiology Panel

Anna Cassoni, Radiotherapy Panel

Denise Blake, Pharmacy Panel

(*) Group representative

### A4. Scandinavian Sarcoma Group (SSG)

#### Norway.

Oslo University Hospital (Dr Kirsten Sundby Hall); Bergen University Hospital (Dr Odd Monge); Trondheim University Hospital (Dr Erling Moe).

#### Sweden.

Umeå University Hospital (Dr Ulf Hjalmars (Children)Dr Beatrice Malmer, Dr Kjell Johansson (Children)); Linköping University Hospital (Dr Najme Wall, Dr Maria Östlund, Dr Mikael Behrendtz); Gothenburg Sahlgrenska University Hospital (Dr Lina Hansson, Dr Gustaf Österlundh (Children), Dr Monika Sender, Dr Katarina Engström,); Lund University Hospital, Oncologic dept. (Dr Mikael Eriksson, Dr Lars Hjorth (Children)); Akademiska sjukhuset, Uppsala University Hospital (Dr Ingela Turesson, Dr Gustaf Ljungman (Children)); Karolinska University Hospital, Stockholm (Dr Elisabet Lidbrink, Dr Cecilia Petersen (Children), Dr Mikael Szeps, Dr Annika Folin, Dr Christina Linder-Stragliotto, Dr Jonas Karlén (Children), Dr Åke Jacobson (Children)).

#### Finland.

Tampere University Hospital (Dr Tuula Lehtinen); Helsinki University Central Hospital (Dr Maija Tarkkanen); Turku University Hospital (Dr Paula Lindholm).

#### Denmark.

Aarhus University Hospital (Dr Akmal Safwat (Adult), Dr Ole Steen Nielsen, Dr Henrik Hasle (Children)); Copenhagen Rigshospital (Dr Catherine Rechnitzer).

### SSG Panel Representatives and Additional Contributors

Sigbjørn Smeland, Chief Investigator; TMG Member: Chief Investigator; National Coordinator: Norway Oncology Panel

Maria Rejmyr-Davis, TMG Member: Data Manager

Eva-Mari Olofsson, TMG Member: Research Administrator

Elisabeth Johansson, TMG Member: Data Manager

Linda Werner-Hartman, TMG Member: Statistician

Thor Alvegård, TMG Member: Data Manager Supervisor

Ole Sten Nielsen, National Coordinator: Denmark

Maija Tarkkanen, National Coordinator: Finland

Oskar Johansson, National Coordinator: Iceland

Mikael Eriksson, National Coordinator: Sweden

Oskar Hagberg, Statistician

Karolina Carlsson, Statistician

Viktoria Samuelsson, Statistician

Anna Bladtröm, Statistician

Jeanette Ceberg, Monitor

Christina Danewid, Monitor

Mercedes Marotta, Data Systems Engineer

Mona Malström, CRF Designer

**Oncology Panel:**

Thomas Wiebe, Oncology Panel

Åke Jakobson, Oncology Panel

Maija Tarkkanen, Oncology Panel

Ole Steen Nielson, Oncology Panel

Tom Böhling,* Pathology Review Panel

Lars-Gunnar Kindblom,* Pathology Review Panel

Henryk Domanski, Pathology Review Panel

Bodil Bjerkehagen, Pathology Review Panel

Johan Wejde, Pathology Review Panel

Ola Myklebost, Biological Studies Panel

Nils Mandahl, Biological Studies Panel

Sakari Knuutila, Biological Studies Panel

Otte Brosjö, Surgical Panel

Ingeborg Taksdal,* Radiology Panel

Veli Söderlund, Radiology Panel

Øyvind Bruland, Radiotherapy Panel

Linda Werner-Hartman, Statistics Panel

Tor Skärby, Pharmacy Panel

(*) Group representative

### A5. Members of Independent Oversight Committees

**Independent Data Monitoring Committee:**

Barry Hancock, Chair: Sheffield, United Kingdom

Gerald Gilchrist, Member: Minnesota, United States

Otilia Dalesio, Member: Amsterdam, the Netherlands

Peter Høglund, Member: Lund, Sweden

**Trial Steering Committee:**

Stefano Ferrari, Chair: Bologna, Italy

Joseph Mirro, Member: Memphis, United States

Hans Strander, Member: Stockholm, Sweden

Robert Souhami, Member: London, United Kingdom

### A6. Members of EURAMOS Intergroup Safety Desk (EISD)

Trude Butterfaß-Bahloul, TMG Member: Clinical Research Associate SAE, Safety Desk Manager

Heidi Oellers, TMG Member: Monitoring/Auditing

Marc Urban, TMG Member: Monitoring/Auditing

Karl-Friedrich Lukat, Clinical Research Associate SAE, Safety Desk Manager

Melanie Langeleist: Safety Desk Assistant

Dorothe Hülser: Safety Desk Assistant

Gudrun Würthwein: Data Management of Safety Database

Sonja Baier: Data Management of Safety Database

Attyla Drabik: Safety Desk Manager

Charlotte Young: Safety Desk Manager

Kirsten Werner: Safety Desk Manager

Andrea Paneitz: Safety Desk Manager

Ruth Wagner: Safety Desk Manager

Eva Grünewald: Safety Desk Manager

Christiana Rohde-Osei: Safety Desk Assisant

Kerstin Hovestadt: Safety Desk Assistant

Linus Lauterbach: Safety Desk Assistant

### B7. Members of Quality of Life Coordinating Centre

Gabriele Calaminus, TMG Member: Quality of Life Panel

Andreas Wiener, TMG Member: Quality of Life Panel

Katja Baust: Psychotherapist

Carmen Teske: Study Documentation

Karina Riemenschneider: Secretary
