# ERRATA

**DOI:** 10.1200/JCO.2016.70.9923

**Published:** 2016-11-20

The July 10, 2015, article by Bielack et al entitled “Methotrexate, Doxorubicin, and Cisplatin (MAP) Plus Maintenance Pegylated Interferon Alfa-2b Versus MAP Alone in Patients With Resectable High-Grade Osteosarcoma and Good Histologic Response to Preoperative MAP: First Results of the EURAMOS-1 Good Response Randomized Controlled Trial,” (J Clin Oncol 33:2279-2287, 2015) contained errors.

In Figure 1, the dosing for both cisplatin and doxorubicin was given incorrectly. It was given as “120 g/m^2^” and “75 g/m^2^,” respectively, but should read as “120 **m**g/m^2^” and “75 **m**g/m^2^.”

Also, in the Results section under the Treatment subheading, the target dosage for cisplatin was written as “240 mg/^2^.” This should be “240 mg/**m**^2^.”

The online version has been corrected in departure from the print. *Journal of Clinical Oncology* and the authors apologize for the errors.

